# North American House Sparrows Are Competent for Usutu Virus Transmission

**DOI:** 10.1128/msphere.00295-22

**Published:** 2022-11-01

**Authors:** Sarah C. Kuchinsky, Jeffrey Marano, Seth A. Hawks, Emma Loessberg, Christa F. Honaker, Paul B. Siegel, Chloé Lahondère, Tanya LeRoith, James Weger-Lucarelli, Nisha K. Duggal

**Affiliations:** a Department of Biomedical Sciences and Pathobiology, Virginia-Maryland College of Veterinary Medicine, Virginia Polytechnic Institute and State University, Blacksburg, Virginia, USA; b Department of Animal and Poultry Sciences, Virginia Polytechnic Institute and State University, Blacksburg, Virginia, USA; c Department of Biochemistry, Virginia Polytechnic Institute and State University, Blacksburg, Virginia, USA; d Department of Entomology, Virginia Polytechnic Institute and State University, Blacksburg, Virginia, USA; e Center of Emerging, Zoonotic and Arthropod-borne Pathogens, Virginia Polytechnic Institute and State University, Blacksburg, Virginia, USA; f The Global Change Center, Virginia Polytechnic Institute and State University, Blacksburg, Virginia, USA; Stanford University School of Medicine

**Keywords:** arbovirus, house sparrow, mosquito, transmission

## Abstract

Usutu virus (USUV, *Flaviviridae*) is an emerging mosquito-borne virus that has been implicated in neuroinvasive disease in humans and epizootic deaths in wild birds. USUV is maintained in an enzootic cycle between ornithophilic mosquitoes, primarily *Culex* spp., and wild birds, predominantly passerine species. However, limited experimental data exist on the species competent for USUV transmission. Here, we demonstrate that house sparrows are susceptible to multiple USUV strains. Our study also revealed that Culex quinquefasciatus mosquitoes are susceptible to USUV, with a significantly higher infection rate for the Netherlands 2016 USUV strain compared to the Uganda 2012 USUV strain at 50% and 19%, respectively. To assess transmission between avian host and mosquito vector, we allowed mosquitoes to feed on either juvenile chickens or house sparrows inoculated with USUV. Both bird models transmitted USUV to *C. quinquefasciatus* mosquitoes. Linear regression analyses indicated that *C. quinquefasciatus* infection rates were positively correlated with avian viremia levels, with 3 to 4 log_10_ PFU/mL representing the minimum avian viremia threshold for transmission to mosquitoes. Based on the viremia required for transmission, house sparrows were estimated to more readily transmit the Netherlands 2016 strain compared to the Uganda 2012 strain. These studies provide insights on a competent reservoir host of USUV.

**IMPORTANCE** Usutu virus (USUV) is a zoonotic mosquito-borne virus that can cause neuroinvasive disease, including meningitis and encephalitis, in humans and has resulted in hundreds of thousands of deaths in wild birds. The perpetuation of USUV in nature is dependent on transmission between *Culex* spp. mosquitoes and various avian species. To date, few experimental data exist for determining which bird species are important for the maintenance of USUV. Our studies showed that house sparrows can transmit infectious Usutu virus, indicating their role as a competent host species. By identifying reservoir species of USUV, we can predict areas of USUV emergence and mitigate its impacts on global human and wildlife health.

## INTRODUCTION

Usutu virus (USUV) is an emerging mosquito-borne virus that requires an ornithophilic mosquito vector, primarily *Culex* spp., and an avian host for its viral maintenance (1, 2). As a member of the Japanese encephalitis serocomplex of the *Flaviviridae* family, USUV shares similar antigenic properties with its other members, such as West Nile virus (WNV) and St. Louis encephalitis virus (SLEV) (3–5). Originally isolated in South Africa in 1959 (6), USUV is now emerging in Europe (7). USUV is zoonotic and is associated with neuroinvasive disease in humans. Acute USUV infections in humans have been reported in approximately 80 individuals in Europe and Africa (8–21).

Not only is USUV of significance for human public health, but it is also a major concern for wildlife health. USUV causes disease in wild bird populations, where the burden of death is greatest in the Eurasian blackbird (*Turdus merula*). The earliest indication of USUV circulating in European wild bird populations occurred in 2001 in Austria (22), though retrospective analysis suggests that USUV was circulating 5 years earlier in Italy (23). Since its establishment in Europe, USUV has been implicated in the decline of breeding bird populations (24, 25). In epizootic events between 2003 and 2012, blackbird die-offs ranged from roughly 1,000 in Italy (26) to approximately 50,000 in Austria (27), and to upwards of 400,000 in Germany (24). Deaths have also been reported in great gray owls (*Strix nebulosa*) (25) and house sparrows (*Passer domesticus*) (28). While it is apparent that birds in the orders Passeriformes or Strigiformes are more vulnerable to a fatal outcome, USUV has been detected in birds from 17 additional orders, representing over 100 different species found in Africa or Europe (1, 15, 29–32). Although USUV infection has been reported in a diverse array of species, the species which serve as reservoir hosts, critical for viral transmission, have yet to be determined.

Most studies documenting naturally infected mosquitoes in Africa and Europe have predominantly reported on *Culex* spp. mosquitoes, including *C. neavei* (33), *C. modestus* (34), *C. perexiguus* (35), *C. univitattus* (36), and *C. pipiens* (37, 38). USUV has also been detected in *Aedes* spp. (2), *Ochlerotatus* spp. (37), and *Mansonia* spp. (6) mosquitoes. European colonies of *C. pipiens* were shown to be highly competent for USUV when fed an infectious bloodmeal with the Bologna 2009 or Netherlands 2016 USUV strains (39, 40). *C. pipiens* and *C. quinquefasciatus* colonies from North America which fed on an infectious bloodmeal with the prototype USUV strain, South Africa 1959, were both susceptible to infection (41). Another study using a South African lab colony of *C. quinquefasciatus* reported transmission in saliva (42). USUV infection and transmission has also been described in other mosquito species, including *C. neavei* (43) and *Aedes japonicus* (44). While numerous studies have shed light on the important potential vector species competent for USUV using artificial bloodmeals, none to date have assessed their infection potential following exposure to an infected avian host.

Passerine species were identified as the major reservoir hosts for WNV and SLEV through experimentation demonstrating transmission from viremic birds to *Culex* spp. mosquitoes (45, 46). Due to the challenges associated with assessing viral transmission from wild avian species to mosquitoes, limited experimental data exist and thus models have been developed based on one or two species to estimate the likelihood that other avian species will serve as a reservoir host, per the reservoir competence index (45, 47). In particular, juvenile chickens have been used as models in flavivirus transmission experiments to determine the minimum infectious threshold of transmitting virus from avian host to mosquito (46, 48, 49). The reservoir competence index (*C*) is the product of three components: host susceptibility to infection (*s*); host infectiousness (*i*), or the estimated proportion of mosquitoes that become infected after feeding on a host; and the duration of host infectiousness (*d*), or how long the host sustains an infectious viremia level (50). Based on these studies, house sparrows and other passerine species have been defined as competent reservoir species for WNV (45).

The aim of this study was to determine whether North American house sparrows are competent for USUV. The house sparrow, an invasive species in the United States that was originally introduced from Europe, is a competent host for WNV (45). House sparrows experimentally inoculated with multiple strains of USUV were susceptible and reached high viremia levels. To determine the level of avian viremia required for transmission, *C. quinquefasciatus* mosquitoes were fed upon USUV-infected juvenile chickens or house sparrows. USUV was transmitted from birds to mosquitoes, with significantly higher predicted mosquito infection rates for a European USUV strain compared to an African USUV strain. These experiments provide much needed insights on identifying reservoir host species of USUV.

## RESULTS

### Susceptibility of *C. quinquefasciatus* mosquitoes to USUV.

**FIG 1:**
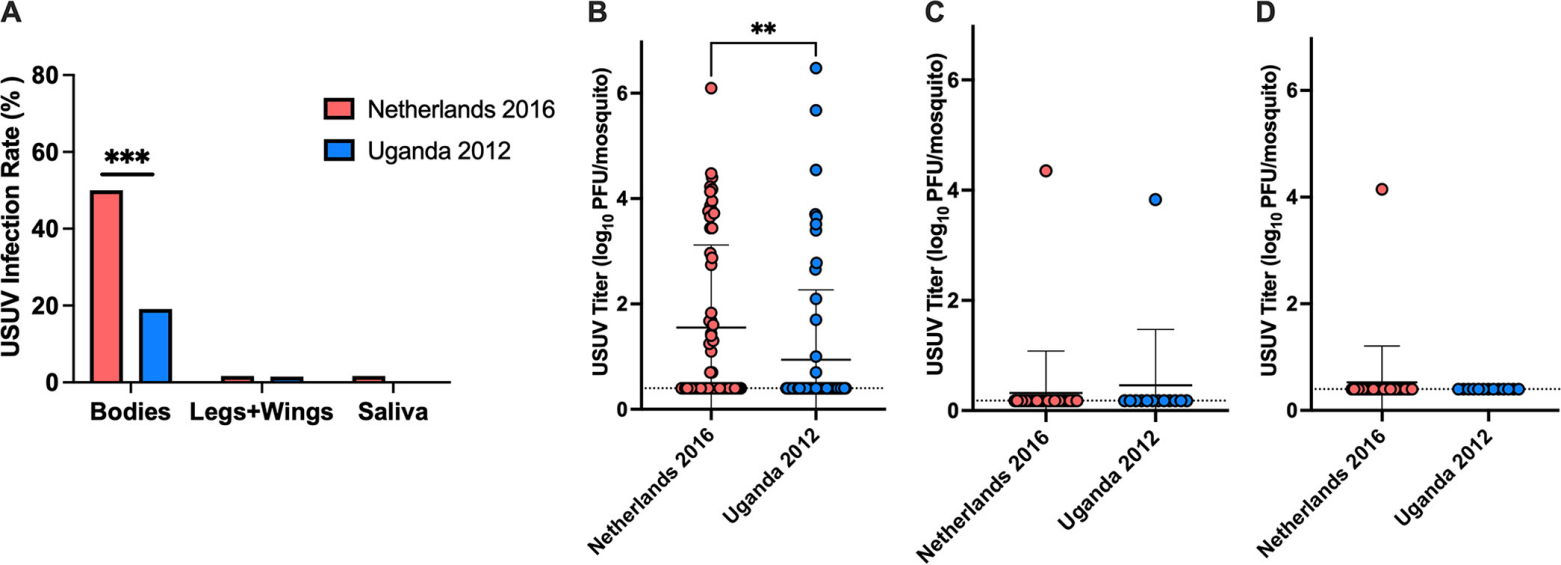
Culex quinquefasciatus mosquitoes are susceptible to Usutu virus (USUV) infection. (A) Proportion of mosquitoes found positive for infectious USUV out of the total number of mosquitoes that were fed on a bloodmeal containing the Netherlands 2016 or Uganda 2012 USUV strain. Mosquito bodies represent infection rates; legs and wings represent dissemination rates; saliva represents transmission rates. Fisher’s exact test was used to compare proportions. ***, *P < *0.001. (B) Viral titers in mosquito bodies. (C) Viral titers in mosquito legs and wings. (D) Viral titers in mosquito saliva. Circles represent individual samples; horizontal lines represent the mean; error bars represent standard deviation. Limit of detection (LOD) is represented by the dashed line. Mann-Whitney test was used to compare viruses. **, *P < *0.01.

The susceptibility and vector competence of *C. quinquefasciatus* for USUV was evaluated through artificial bloodmeals spiked with either Netherlands 2016 or Uganda 2012 USUV strains. Bloodmeals were back-titrated, indicating that mosquitoes were exposed to 7.5 and 7.4 log_10_ PFU/mL for Netherlands 2016 and Uganda 2012, respectively. Fifty percent (30/60) of mosquitoes exposed to Netherlands 2016 were infected, a significantly higher proportion compared to the 19% (13/68) of mosquitoes that were infected following exposure to Uganda 2012 (Fisher’s exact test, *P = *0.0003, Fig. 1A). Additionally, Netherlands 2016-exposed mosquitoes had significantly higher titers in body homogenates relative to Uganda 2012-exposed mosquitoes (*P = *0.0045, Fig. 1B). Both groups had comparable dissemination rates: 1.7% (1/60) of mosquitoes exposed to Netherlands 2016 and 1.5% (1/68) of those exposed to Uganda 2012 had detectable virus in the legs/wings (Fig. 1A) with similar titers (Fig. 1C). Limited transmission potential in saliva was observed. However, infectious virus was detected in one saliva sample (1.7%) from a Netherlands 2016-exposed mosquito, with a titer of 4.1 log_10_ PFU/mosquito, but not in Uganda 2012-exposed mosquito saliva samples (0/68) (Fig. 1A and D). Together these data indicate that *C. quinquefasciatus* mosquitoes are susceptible to USUV infection, though only a weakly competent vector.

### A chicken model for USUV enzootic transmission.

**FIG 2:**
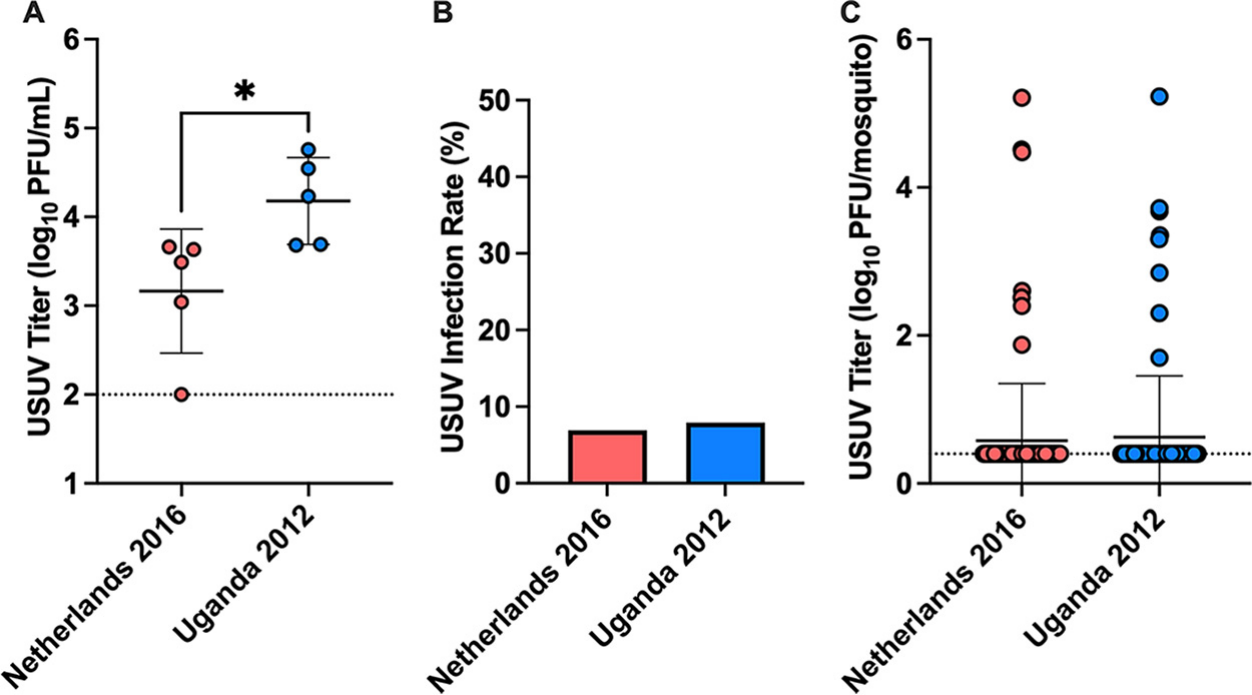
Low antibody response (LAS) chicken model transmits USUV to *C. quinquefasciatus* mosquitoes. (A) Viremia in LAS chicks inoculated with Netherlands 2016 or Uganda 2012 USUV strain. Circles represent individual samples; horizontal lines represent the mean; error bars represent standard deviation. Limit of detection is represented by the dashed line. Mann-Whitney test was used to compare viruses. *, *P < *0.05. (B) Proportion of mosquito bodies positive for infectious USUV out of the total number of mosquitoes fed on either Netherlands 2016-inoculated or Uganda 2012-inoculated LAS chicks. Fisher’s exact test was used to compare proportions; no significance was observed. (C) USUV titers in mosquito bodies fed on USUV-inoculated LAS chicks. Circles represent individual samples; horizontal lines represent the mean; error bars represent standard deviation. Limit of detection is represented by the dashed line. Mann-Whitney test was used to compare viruses; no significance was observed.

To assess USUV transmission between *C. quinquefasciatus* and an avian host, mosquitoes were fed on individual low antibody response (LAS) chicks inoculated 2 days prior with 1,500 PFU USUV, an avian model of infection previously established by our lab (51). Chicks inoculated with Netherlands 2016 developed significantly lower viremia on 2 days postinoculation (dpi) than those inoculated with Uganda 2012, with means of 3.2 and 4.2 log_10_ PFU/mL, respectively (*P = *0.0286, Fig. 2A). Transmission of USUV from chicks to mosquitoes was evident, with infectious virus detected in 6% (7/116) of mosquitoes that fed upon Netherlands 2016-infected birds and 8% (8/101) of those that fed upon Uganda 2012-infected birds (Fig. 2B). There was no significant difference in the mean titer of mosquito body homogenates between virus strains (Fig. 2C).

### House sparrows are susceptible to African and European USUV strains.

**FIG 3:**
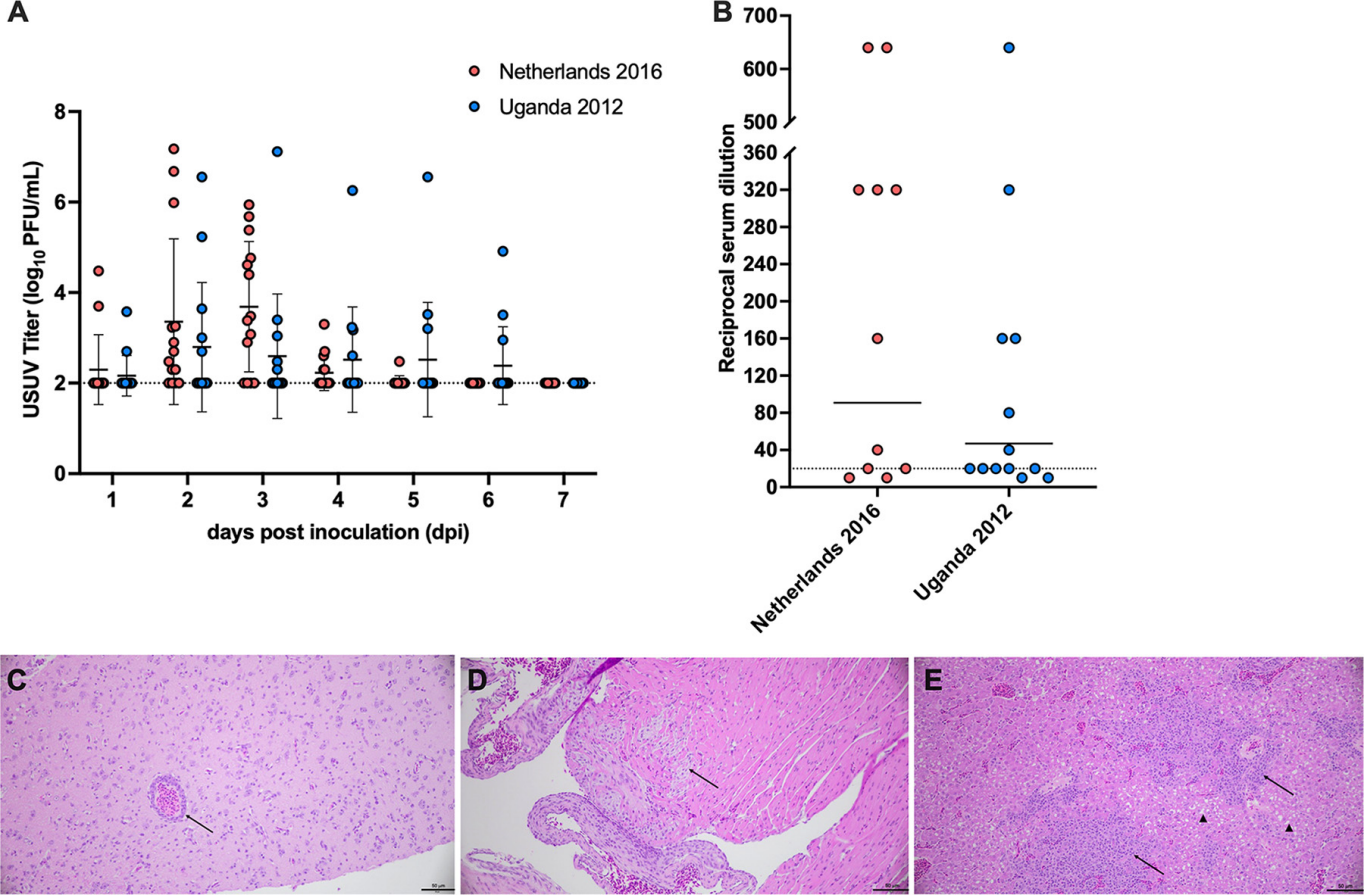
House sparrows are susceptible to African and European USUV strains. (A) Viremia in house sparrows inoculated with Netherlands 2016 or Uganda 2012 USUV strain. Circles represent individual samples; horizontal lines represent the mean; error bars represent standard deviation. Limit of detection is represented by the dashed line. Multiple Mann-Whitney tests using the Holm-Sidak method were used to compare viruses; no differences were observed. (B) Neutralizing antibody response was measured by plaque reduction neutralization test (PRNT); samples were considered positive if they reached the 90% reduction threshold (PRNT_90_). Highest reciprocal serum titer where 90% reduction was reached is shown; circles represent individual serum samples. The geometric mean titer (GMT) of each group is represented by a solid line. Samples that did not reach 90% reduction threshold were considered negative and graphed at half-LOD, 10. A Mann-Whitney test was used to compare GMTs; no significance was observed. (C) Brain tissue collected on 14 dpi from house sparrow inoculated with Netherlands 2016 USUV strain, with perivascular cuffing (arrow) (hematoxylin and eosin [H&E] stain). (D) Heart tissues collected on 14 dpi from house sparrow inoculated with Uganda 2012 USUV strain, with foci of inflammation and myocardial degeneration (arrow) (H&E stain). (E) Liver tissue from house sparrow inoculated with Uganda 2012 USUV strain, with lesions of lymphocytic inflammation (arrow) and hepatic degeneration (arrowhead) (H&E stain). Scale bars = 50 μm.

To better understand enzootic transmission of USUV, we established another avian model of infection using house sparrows. Wild-caught house sparrows, confirmed to be seronegative for WNV, were divided into two groups (*n *=* *14) and subcutaneously inoculated with 1,500 PFU of USUV strain Netherlands 2016 or Uganda 2012. Viremia was measured daily for 7 days following inoculation; 89.3% of house sparrows became viremic. The mean peak titer for the Netherlands 2016-inoculated group occurred at 3 dpi and was 3.7 log_10_ PFU/mL (Fig. 3A). The mean peak titer for the Uganda 2012-inoculated group occurred on 2 dpi and was 2.8 log_10_ PFU/mL. Multiple individuals in both experimental groups had titers at or above 5 log_10_ PFU/mL at multiple time points. At 6 dpi, one individual from the Uganda 2012-inoculated group exhibited clinical signs of illness, including ruffled feathers, lethargy, and poor responsiveness, and was euthanized immediately.

A final blood sample was collected on 14 dpi to assess for seroconversion by plaque reduction neutralization test (PRNT). One Uganda 2012-inoculated house sparrow never developed viremia, but did neutralize USUV at a titer of 20, indicating exposure to the virus. Thus, we observed a 92.9% infection rate in house sparrows. There was no significant difference in the 90% PRNT (PRNT_90_) titers between strains (Fig. 3B). These data indicate that, when exposed to USUV, house sparrows develop a robust neutralizing antibody response.

Tissue samples were collected from a subset of birds on 14 dpi to evaluate for microscopic evidence of USUV infection. Perivascular cuffing was observed in the brain of one house sparrow inoculated with the Netherlands 2016 USUV strain (Fig. 3C). A region of inflammation, as evidenced by lymphocytic infiltrates and myocardial degeneration, was observed in the heart tissue of a bird inoculated with the Uganda 2012 USUV strain (Fig. 3D). Multifocal lymphocytic infiltrates and hepatic degeneration were observed in a Uganda 2012-inoculated house sparrow (Fig. 3E). Together, these results demonstrate that house sparrows may be an appropriate model to investigate enzootic transmission between an avian host and mosquitoes.

### House sparrows are competent hosts of USUV and exhibit a higher predicted mosquito infectiousness for a European USUV strain.

**FIG 4:**
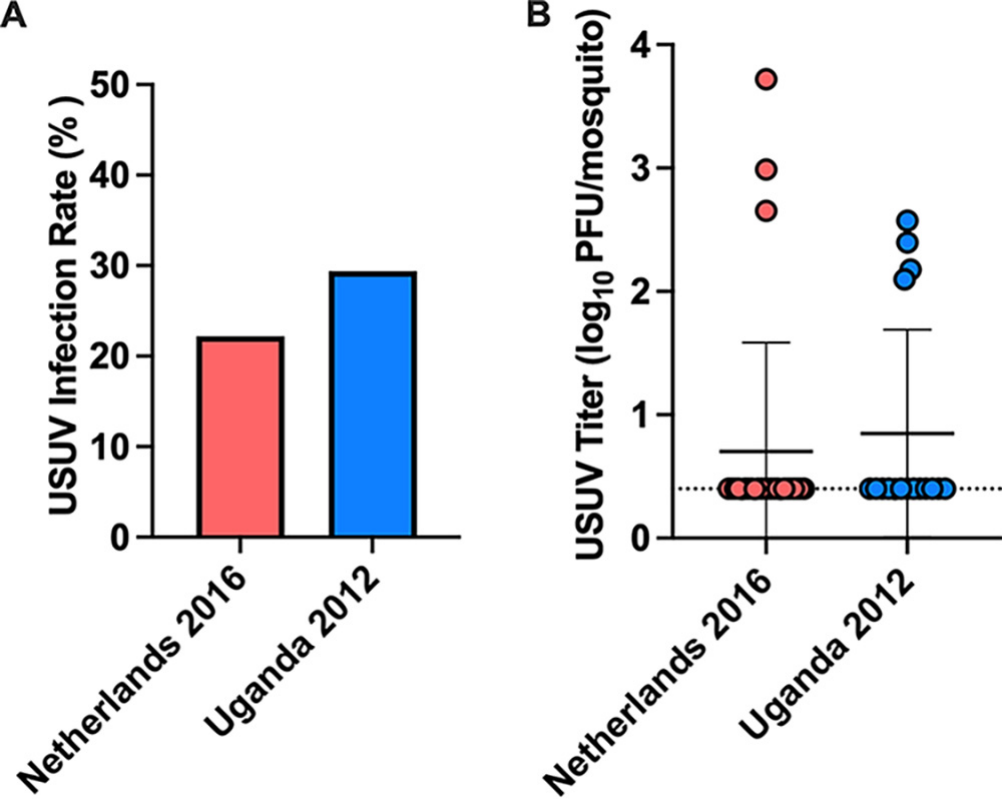
House sparrows transmit USUV to *C. quinquefasciatus* mosquitoes. (A) Proportion of mosquito bodies positive for USUV out of the total number of mosquitoes fed on either Netherlands 2016-inoculated or Uganda 2012-inoculated house sparrows. Fisher’s exact test was used to compare proportions; no significance was observed. (B) USUV titers in mosquito bodies fed on USUV-inoculated house sparrows. Circles represent individual samples; horizontal lines represent the mean; error bars represent standard deviation. Limit of detection is represented by the dashed line. Mann-Whitney test was used to compare viruses; no significant difference was observed.

In a similar experimental setup to that for the LAS chickens, mosquitoes were fed upon USUV-inoculated house sparrows on 2 dpi to assess USUV transmission. There was a 22.2% (6/27) infection rate in mosquitoes that fed upon Netherland 2016-inoculated house sparrows and a 29.4% (5/17) infection rate in those that fed upon Uganda 2012-inoculated house sparrows (Fig. 4A). No significant difference in the mean titers of mosquito body homogenates was detected between groups, and titers were consistent with the mean body titers of mosquitoes that fed on LAS chicks (Fig. 4B). These results indicate that the house sparrow is a competent species for USUV transmission.

**FIG 5:**
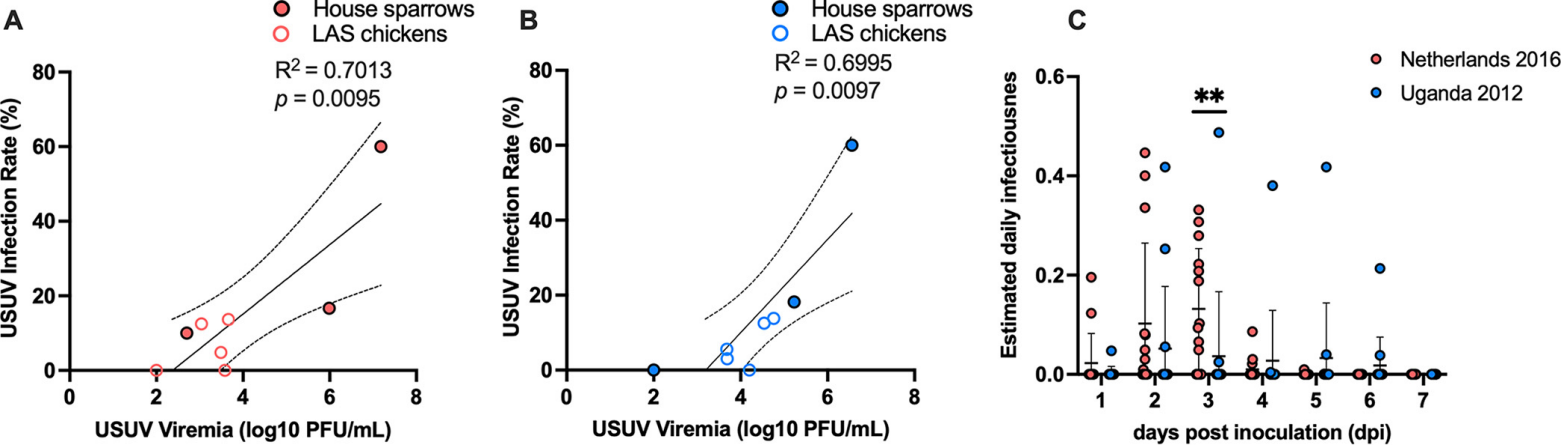
Estimated USUV infectiousness relative to avian viremia level. (A) Linear regression analysis of Netherlands 2016 avian viremia titer (log_10_ PFU/mL) compared to *C. quinquefasciatus* mosquito USUV infection rates. (B) Linear regression analysis of Uganda 2012 avian viremia titer (log_10_ PFU/mL) compared to *C. quinquefasciatus* mosquito USUV infection rates. Open circles represent mosquitoes that fed on LAS chicks; closed circles represent mosquitoes that fed on house sparrows. Dashed lines represent 95% confidence intervals. (C) Estimated daily infectiousness of house sparrows inoculated with either Netherlands 2016 or Uganda 2012 over the course of 7 days. Circles represent individual samples; horizontal lines represent the mean; error bars represent standard deviation. Multiple Mann-Whitney tests using the Holm-Sidak method were used to compare viruses. **, *P < *0.01.

To determine whether there was a correlation between USUV titers in avian blood samples and mosquito infection rates, a linear regression analysis was performed (see Materials and Methods). Chicken and house sparrow data were grouped by virus strain to compare avian viremia titers with their respective mosquito infection rates. For both virus strains, we found a positive relationship between avian viremia titer and mosquito infection rate (Fig. 5A, *P = *0.0095; Fig. 5B, *P = *0.0097). Furthermore, we observed that birds with a minimum of 2.7 log_10_ PFU/mL viral titer and those with a minimum of 3.7 log_10_ PFU/mL viral titer were capable of transmitting Netherlands 2016 and Uganda 2012, respectively, to *C. quinquefasciatus* mosquitoes, although this difference was not significant (*P = *0.44). Together, these data suggest that avian viremia levels play a crucial role in determining the proportion of infected vectors and ultimately the likelihood of USUV transmission.

The estimated daily mosquito infectiousness, or the proportion of mosquitoes that are predicted to become infected as a result of feeding on a host, was calculated for all house sparrows based on the linear regression equation (45, 47). There was a significantly higher estimated daily infectiousness for Netherlands 2016-inoculated house sparrows compared to Uganda 2012-inoculated house sparrows on 3 dpi (Fig. 5C, *P = *0.0036). This indicates that, when applied to animals on a population level, Uganda 2012-inoculated house sparrows are predicted to infect fewer mosquitoes due to lower average viremias. To further understand the role of house sparrows as USUV reservoir hosts, we calculated the overall reservoir competence index (the product of host susceptibility, host infectiousness, and duration of host infectiousness) (50). The reservoir competence index was 0.27 for house sparrows inoculated with Netherlands 2016 and 0.17 for those inoculated with Uganda 2012. This indicates that 27% of mosquitoes that feed on house sparrows infected with Netherlands 2016 are expected to become infected, while 17% of mosquitoes that feed on house sparrows infected with Uganda 2012 are expected to become infected.

## DISCUSSION

Here, we showed that house sparrows are susceptible to multiple strains of USUV and can transmit it to *Culex* spp. mosquitoes, indicating their potential role as a reservoir host for the virus. Moreover, we showed that a 2.7 to 3.7 log_10_ PFU/mL viremia was sufficient to transmit USUV to *C. quinquefasciatus* mosquitoes. Finally, we found that house sparrows infected with the Netherlands 2016 strain are predicted to be more infectious to mosquitoes than house sparrows infected with Uganda 2012 strain.

Our results suggest that the house sparrow has the potential to serve as a reservoir host for USUV due to its susceptibility to infection and ability to transmit multiple USUV strains to *Culex* spp. mosquitoes. USUV infection has been reported in naturally infected house sparrows in Austria (52), Switzerland (28), and recently in the United Kingdom, where viral antigen was detected in neurons and endothelial cells in the brain and in renal tubular epithelial cells in the kidney (53). In our study, microscopic evidence of USUV disease in house sparrows, including inflammatory and immune cell infiltrates in the brain, heart, and liver, is consistent with reports of disease in USUV-positive wild birds in Europe (28, 54, 55). Therefore, experimentally inoculated house sparrows present similar signs of disease as naturally infected house sparrows. Additionally, the lack of clinical signs in our experimentally inoculated house sparrows is supportive of reservoir host potential. Furthermore, house sparrows have been implicated as epidemiologically important species for SLEV and WNV (56, 57). With *Culex* spp. mosquitoes serving as competent vectors for USUV (39) and house sparrows serving as a common bloodmeal source of *Culex* mosquitoes in the United States (58–60), it is possible that house sparrows could maintain USUV transmission in locations where *Culex* spp. are present. Thus, we have identified the house sparrow as a relevant USUV reservoir species.

Our study demonstrated a significant relationship between mosquito infection rate and viral load in avian host blood. This correlation has been demonstrated in other bird-mosquito enzootic transmission systems. For example, the infection rates of *C. pipiens* fed upon WNV-inoculated juvenile chickens (48), *C. tarsalis* fed upon WNV-inoculated house sparrows, and *C. quinquefasciatus* fed upon SLEV-inoculated juvenile chickens (46) were dependent on avian viremia titers. Additionally, artificial bloodmeal titers have a dose-dependent effect on *Culex* spp. mosquito infection rates, as has been reported for USUV (43), WNV (46, 61), and SLEV (46). It is evident that viral blood titer influences the proportion of *Culex* spp. mosquitoes which acquire infection. One study demonstrated that the proportion of infected mosquitoes was typically greater after feeding on live avian hosts compared to that after feeding on an artificial bloodmeal, at similar or even lower titers (46), which is consistent with our study. One caveat to our study is that we did not measure the transmission potential of USUV in saliva from mosquitoes fed on infected birds. Additional experimentation to determine whether feeding on live birds increases transmission rates in mosquitoes is warranted, which has also been demonstrated by Reisen et al. (46).

Our data suggests that the USUV minimum infectious threshold for *C. quinquefasciatus* mosquitoes is 3 to 4 log_10_ PFU/mL, which is similar to the minimum infectious thresholds of 4 to 5 log_10_ PFU/mL and 2 to 3 log_10_ PFU/mL for WNV (45) and SLEV (46), respectively. However, the competence of house sparrows for USUV appears to be lower than that for WNV (45, 47). We may have underestimated the magnitude of the host competence of house sparrows for USUV due to the use of North American *C. quinquefasciatus* mosquitoes, which have weak vector competence for USUV. Other groups have also found low vector competence for *C. quinquefasciatus* mosquitoes (41, 42). Other mosquitoes that are more competent for USUV should be tested in future studies. For example, while we observed a 1.7% transmission rate in *C. quinquefasciatus* mosquitoes feeding on an infectious bloodmeal containing Netherlands 2016 USUV strain, a European *C. pipiens* colony was shown to have a 15% transmission rate feeding on the same strain at a comparable bloodmeal titer (5 × 10^6^ TCID_50_ [50% tissue culture infective dose]/mL) (40). *C. pipiens* feeding on an infectious bloodmeal containing the Bologna 2009 strain demonstrated a much higher transmission rate, with infectious saliva detected in 69% of mosquitoes (39). Using a more competent *Culex* spp. mosquito will be essential in future experiments to obtain an accurate measurement of host competence. To further elucidate the species of importance for USUV maintenance, assessing the susceptibility of other potential host species is necessary. Our minimum infectious threshold can then be applied to additional species to determine reservoir competence. However, there are limitations to all models: for example, a minimum infectious threshold model may underestimate the impact abundant hosts with lower viremia levels have on the maintenance of virus in the environment (62). Nonetheless, identifying potential reservoir species serves as a tool for predicting possible spread and emergence of USUV.

By comparing virus strains from Europe and Africa, our results can shed light on the possible impact of viral evolution on transmission. A significant difference between the estimated mosquito infectiousness on 3 dpi was observed between viruses in house sparrows, suggesting that Netherlands 2016-infected house sparrows are more infectious to *C. quinquefasciatus* on 3 dpi than Uganda 2012-infected house sparrows. Other flaviviruses have evolved to increase potential mosquito infectiousness in birds, including WNV, as evidenced by increased viremia in American crows (*Corvus brachyrhynchos*) (63, 64) and house sparrows (65), and Japanese encephalitis virus, as evidenced by increased viremia in poultry (66). Our group has previously identified 23 amino acid differences between these two USUV strains (67). Many of the amino acid distinctions occur in the structural proteins, with 8 found in the envelope protein. Residues in the envelope protein have been characterized as virulence factors for numerous flaviviruses (68–70). However, a recent experiment that designed chimeric viruses using segments of the structural and non-structural genes of WNV and SLEV found that the structural genes were not key in regulating differences in virulence and replication in multiple bird species (71). As such, nonstructural genes have also been implicated as virulence factors in flaviviruses (72). Netherlands 2016 and Uganda 2012 also had amino acid differences in NS1, NS2A, NS3, NS4B, and NS5. A previous study demonstrated that at least one site in the NS1, NS3, and NS5 genes in USUV had undergone positive selection (73). Therefore, determining which amino acid differences account for increased viremia is an important avenue of research to explore in order to understand flavivirus emergence and USUV genetic determinants of transmission.

Understanding the geographic range of house sparrows and *Culex* spp. mosquitoes is essential to predicting where potential USUV transmission may occur. Native to Europe, Asia, and North Africa, house sparrows are now found on every continent except Antarctica and are considered the most widely distributed bird species (74, 75). Mosquitoes in the *C. pipiens* complex, including *C. pipiens* and *C. quiquefasciatus*, are also found on every continent except Antarctica, with *C. pipiens* being the predominant species in Europe, *C. quinquefasciatus* predominating in South America, and a mix of both species occuring in North America, Africa, and Asia (76). As such, house sparrows and mosquitoes in the *C. pipiens* complex are ubiquitous and coupled with the ever-increasing occurrence of flaviviruses across the globe (77), it will be vital to continue monitoring USUV transmission globally.

## MATERIALS AND METHODS

### Viruses and cells.

The following USUV isolates were used throughout these experiments: TMNetherlands (Netherlands 2016, Europe 3 lineage, MN813490, passage 5, isolated from *Turdus merula*) (25) and UG09615 (Uganda 2012, Africa 3 lineage, MN813491, passage 3 to 4, isolated from *Culex* spp.) (36). These strains were previously sequenced by our laboratory (67, 78). Vero cells were cultured in Dulbecco’s modified Eagle medium (Corning) supplemented with 5% fetal bovine serum (FBS, VWR International) and 1% penicillin-streptomycin (Gibco); cells were maintained at 37°C with 5% CO_2_.

### Mosquito experiments.

**(i) Mosquito rearing.** Culex quinquefasciatus (Say) came from a laboratory colony originally collected in Sebring County, Florida in 1988 (79). Larvae were reared on Tropical First Bites fish food (Hikari Sales USA, Inc.). Adult mosquitoes were provided 10% sucrose *ad libitum* for maintenance or defibrinated sheep blood (Colorado Serum Company) and 50% sucrose (5% sucrose in total volume) for egg production. Mosquitoes were maintained in an environmental chamber at 26°C to 27°C on a 16:8 light:dark cycle at 60% to 70% relative humidity.

**(ii) Artificial infectious blood feeds.** Adult *C. quinquefasciatus* mosquitoes, 6 to 8 days post-emergence, were sorted into cartons with 50 females and 10 males. Mosquitoes were starved for 16 to 18 h prior to an infectious bloodmeal, which was dispensed onto a cotton ball. Mosquitoes were exposed to the cotton ball with 3 mL of blood containing 7.5 and 7.4 log_10_ PFU/mL of Netherlands 2016 or Uganda 2012, respectively, for 14 to 16 h. Engorged mosquitoes were sorted under cold anesthesia and returned to the carton; unfed mosquitoes were discarded. Mosquitoes were maintained for 14 days with 10% sucrose *ad libitum*. On day 14 post-exposure, surviving mosquitoes were dissected under cold anesthesia. Legs and wings were removed and stored in 300 μL of mosquito diluent (RPMI 1640 with l-glutamine + 25 mM HEPES [Gibco], 2% FBS [VWR], 50 μg/mL gentamicin [Genesee Scientific], and 2.5 μg/mL amphotericin B [Gibco]). Forced salivation assays (80) were performed as follows. The mosquito proboscis was inserted into the cut end of a pipette tip containing 10 μL of immersion oil. Mosquitoes were salivated for 60 to 80 min. Following salivation, bodies were removed and stored in 500 μL of mosquito diluent, and the salivary excretions were dispensed into 50 μL of mosquito diluent. All mosquito body, leg, wing, and saliva samples were frozen at −80°C until further processing. Artificial blood feed experiments were performed twice independently.

### Chicken experiments.

**(i) LAS chicken inoculations.** The LAS line, previously characterized as susceptible to USUV infection (51), originated from a common White Leghorn founder population and has been selected for >40 generations for a single trait: low antibody response against sheep red blood cells (81, 82). Ten 1-day-old chicks were randomized into two groups. Groups (*n *=* *5) of LAS chicks were subcutaneously inoculated with 1,500 PFU of the Netherlands 2016 or Uganda 2012 USUV strain. A blood sample was collected by jugular venipuncture on day 2 following inoculation, prior to the mosquito feed.

**(ii) Mosquito-LAS chicken infectious feed.** Adult *C. quinquefasciatus* mosquitoes, 7 to 10 days post emergence, were sorted into cartons with 35 to 50 females and 7 to 10 males each. Mosquitoes were starved for 16 to 18 h prior to exposure to USUV-infected chicks.

On 2 dpi, each chick was paired with a carton of mosquitoes. At the time of feeding, chicks were gently restrained against the mesh of the mosquito carton; mosquitoes were allowed to feed on them for 30 min (45, 83). Engorged mosquitoes were sorted under cold anesthesia and returned to the carton. Unfed mosquitoes were discarded. Mosquitoes were maintained for 14 days under previously described conditions. On day 14 post-exposure, surviving mosquito bodies were collected in 500 μL of mosquito diluent and held at −80°C until further processing. Following the mosquito feed, chicks were euthanized by CO_2_ asphyxiation followed by cervical dislocation.

**(iii) Ethics.** All experiments were performed in accordance with the Virginia Tech Institutional Animal Care and Use Committee (IACUC no. 21-048). Throughout the experiments, commercial feed and fresh water were provided *ad libitum*. Chicks were monitored daily for clinical signs by animal care staff and research personnel. If clinical signs including lethargy, ruffled feathers, poor responsiveness, or weight loss of ≥5% were observed, the chick was euthanized via CO_2_ inhalation followed by cervical dislocation.

### House sparrow experiments.

**(i) House sparrow inoculations.** Twenty-eight house sparrows (*Passer domesticus)* of mixed sex and age were trapped with mist nets in Blacksburg, VA during 2019 and 2021. House sparrows were housed at up to 7 birds per cage (76.2 cm length × 45.7 cm width × 45.7 cm height). A blood sample was obtained prior to the start of the experiment to determine previous WNV exposure by plaque reduction neutralization test. In groups of 14, birds were subcutaneously inoculated with 1,500 PFU of USUV strain Netherlands 2016 or Uganda 2012. A blood sample was collected daily for 7 days through jugular venipuncture or lateral wing vein. A final blood sample was collected on 14 dpi, upon which all birds were euthanized. House sparrow inoculations were performed in two independent experiments.

**(ii) Mosquito-house sparrow infectious feed.** Adult *C. quinquefasciatus* mosquitoes, 4 to 12 days post emergence, were sorted into 6 cartons with 50 females and 10 males each. Mosquitoes were starved for 16 to 18 h prior to exposure to USUV-infected house sparrows. There was high overnight mosquito mortality, so group sizes feeding on infected house sparrows were smaller than expected.

On 2 dpi, a subset of house sparrows from each experimental group (*n *=* *3) was paired with a carton of mosquitoes. House sparrows were gently restrained against the mesh of the mosquito carton and mosquitoes were allowed to feed on them for 30 min. Engorged mosquitoes were sorted under cold anesthesia and returned to the carton and unfed individuals were discarded. Mosquitoes were held in the environmental chamber under previously described conditions for 14 days. On day 14 post-exposure, surviving whole mosquitoes were collected in 500 μL of mosquito diluent and held at −80°C until further processing.

**(iii) Ethics.** All experiments were performed in accordance with the Virginia Tech Institutional Animal Care and Use Committee (IACUC no. 21-048) and state scientific collection permit (VADGIF permit no. 070947). Throughout the experiments, commercial wild bird feed supplemented with meal worms and fresh water were provided *ad libitum*. House sparrows were monitored daily for clinical signs by animal care staff and research personnel. If clinical signs such as ruffled feathers, lethargy, or poor responsiveness were observed, the bird was euthanized by CO_2_ inhalation followed by cervical dislocation.

### Viral quantification assays.

Mosquito bodies and legs and wings suspended in mosquito diluent were homogenized in a Qiagen TissueLyserLT at 50 oscillations/sec for 3 min. Samples were clarified by centrifugation at 18,000 rpm for 3 min. Viral titers of serum, mosquito bodies, legs and wings, and saliva were quantified through a Vero cell plaque assay.

### PRNT assays.

Blood collected from house sparrows prior to inoculation and at 14 dpi was assayed by plaque reduction neutralization test. Sera were heat-inactivated at 56°C for 30 min and incubated with approximately 100 PFU of WNV or the homologous USUV strain for 1 h at 37°C before plating on Vero cells. Neutralization activity was defined by plaque reduction at a 90% threshold (84).

### Histopathology.

After euthanasia, a subset of house sparrows was necropsied on 14 dpi to collect tissues, including brain, heart, and liver. Tissues were kept in 10% neutral-buffered formalin prior to routine processing and paraffin embedding. Sections were cut at 5 μm and stained with hematoxylin and eosin for histopathological analysis. Slides were analyzed by a board-certified veterinary pathologist.

### Infectiousness and reservoir competence index.

Using our experimental data from feeding mosquitoes on birds, we calculated a linear regression for the avian blood titer and mosquito infection rate. For Netherlands 2016-inoculated house sparrows, the equation y=9.295x−22.06 was calculated. For Uganda 2012-inoculated house sparrows, the equation y=12.44x−39.77 was calculated. The linear regression equation was then applied to the viremia of all house sparrows to estimate the proportion of *C. quinquefasciatus* mosquitoes predicted to become infected after feeding on each house sparrow, i.e., the estimated daily infectiousness, as described previously (45, 47). The mean estimated daily infectiousness was then determined for each time point (1 to 7 dpi) across all house sparrows, and these values were then summed to calculate the reservoir competence index of house sparrows, as described by Kilpatrick et al. (47).

### Statistical analysis.

Data were checked for normality using the Shapiro-Wilk test. Viral titers were compared using a Mann-Whitney test. House sparrow viremia data and estimated daily infectiousness values were analyzed via multiple Mann-Whitney tests using the Holm-Sidak method to adjust for multiple comparisons. Fisher’s exact test was used to compare mosquito infection, dissemination, and transmission rates. Geometric mean titers were also compared using a Mann-Whitney test. All data was analyzed and graphed using GraphPad Quick Calcs and GraphPad Prism 9 (GraphPad Software, San Diego, CA).
